# Genetic contributions to visuospatial cognition in Williams syndrome: insights from two contrasting partial deletion patients

**DOI:** 10.1186/1866-1955-6-18

**Published:** 2014-07-15

**Authors:** Hannah Broadbent, Emily K Farran, Esther Chin, Kay Metcalfe, May Tassabehji, Peter Turnpenny, Francis Sansbury, Emma Meaburn, Annette Karmiloff-Smith

**Affiliations:** 1 Institute of Education, University of London, London, UK; 2 Birkbeck Centre for Brain and Cognitive Development, University of London, London, UK; 3 Genetic Medicine, St. Mary’s Hospital, Manchester, UK; 4 Royal Devon and Exeter Foundation Trust, Exeter, UK; 5 Penninsula College of Medicine and Dentistry, Universities of Exeter and Plymouth, Exeter, UK

**Keywords:** Williams syndrome, Visuospatial cognition, Navigation, *GTF2I*, *GTF2IRD1*, *LIMK1*

## Abstract

**Background:**

Williams syndrome (WS) is a rare neurodevelopmental disorder arising from a hemizygotic deletion of approximately 27 genes on chromosome 7, at locus 7q11.23. WS is characterised by an uneven cognitive profile, with serious deficits in visuospatial tasks in comparison to relatively proficient performance in some other cognitive domains such as language and face processing. Individuals with partial genetic deletions within the WS critical region (WSCR) have provided insights into the contribution of specific genes to this complex phenotype. However, the combinatorial effects of different genes remain elusive.

**Methods:**

We report on visuospatial cognition in two individuals with contrasting partial deletions in the WSCR: one female (HR), aged 11 years 9 months, with haploinsufficiency for 24 of the WS genes (up to *GTF2IRD1*), and one male (JB), aged 14 years 2 months, with the three most telomeric genes within the WSCR deleted, or partially deleted.

**Results:**

Our in-depth phenotyping of the visuospatial domain from table-top psychometric, and small- and large-scale experimental tasks reveal a profile in HR in line with typically developing controls, albeit with some atypical features. These data are contrasted with patient JB’s atypical profile of strengths and weaknesses across the visuospatial domain, as well as with more substantial visuospatial deficits in individuals with the full WS deletion.

**Conclusions:**

Our findings point to the contribution of specific genes to spatial processing difficulties associated with WS, highlighting the multifaceted nature of spatial cognition and the divergent effects of genetic deletions within the WSCR on different components of visuospatial ability. The importance of general transcription factors at the telomeric end of the WSCR, and their combinatorial effects on the WS visuospatial phenotype are also discussed.

## Background

Williams syndrome (WS) is a rare autosomal dominant disorder arising from the hemizygotic deletion of approximately 27 genes on chromosome 7, at locus 7q11.23 [1,2]. The deletion occurs spontaneously during meiosis and is due to unequal crossing over at misaligned repeat segments [3]. This typically results in a deletion spanning some 1.55 Mb (approximately 95% of cases) to around 1.84 Mb (approximately 5% of cases) of genomic DNA [4-8]. However, a number of individuals with partial deletions within the WS critical region (WSCR) of chromosome 7 have also been identified [for examples, [9,10]. Such cases can provide important insights into the contribution of specific genes to the phenotypic outcome of WS.

Given the uneven cognitive profile characteristic of WS, particularly the contrast between poor non-verbal abilities relative to verbal cognition [for example, [11], research into individuals with partial deletions has sought to identify candidate genes responsible for deficits in domains such as global intellectual difficulties [10], social cognition [12], and spatial cognition [13-15].

In the case of individuals with the full WS deletion, spatial deficits have been well documented, with poor performance reported on visuospatial construction tasks, [16,17], mental imagery [18,19], and the use of spatial frames of reference [20,21]. More recently, deficits in large-scale spatial navigation have also been identified in WS [22,23]. But which genes contribute to these small-scale and large-scale spatial impairments remains a topic of debate. One study that examined two families with a partial WS phenotype, including supravalvular aortic stenosis (SVAS) and deficits in visuospatial construction, found that affected family members were hemizygous for the elastin (*ELN*) and LIM-Kinase1 (*LIMK1*) genes [13], which lie within the WSCR. Given that *ELN* is not expressed in the brain and mutations of which are not associated with spatial deficits but with cardiovascular abnormalities, it was concluded that it must be the other deleted gene, *LIMK1*, that plays an important role in the phenotypic expression of impaired spatial cognition in WS. Indeed, *in vivo*, *Limk1* knockout mice have impaired spatial learning performance when tested on reversal learning in the Morris water maze [24]. They also present with abnormal synaptic structure and neuronal spine morphology, as well as altered hippocampal long-term potentiation.

The role of *LIMK1*, however, has remained inconclusive, with other studies of patients with partial deletions that include *LIMK1* suggesting that hemizygosity for this gene does not in itself result in deficits in visuospatial cognition [for examples., [9,15]. Using a large battery of perceptual and visuospatial tasks, Gray and colleagues [15] report a very detailed assessment of two patients with deletions of only *ELN* and *LIMK1*, compared with two adults with full WS matched on verbal ability. A profile of normal spatial performance emerged from the two partial deletion patients compared to those with the full deletion, suggesting that *LIMK1* alone did not explain spatial deficits in WS. In addition, the successful performance of these same two partial deletion patients on a large-scale spatial task indicated that the hemizygotic deletion of *LIMK1* was also insufficient to result in the poor large-scale search strategies identified in individuals with full WS on the same task [14].

These findings concur to indicate that the sole deletion of *LIMK1* is not sufficient to result in deficits in any form of spatial cognition. Instead, the authors suggest that *LIMK1* may play a role in the spatial cognitive profile in WS only when deleted alongside other genes, particularly those at the telomeric end of the WSCR. It is these latter genes that have been the focus of recent studies.

Hirota *et al*. [25] present a detailed analysis of the relationships between partial deletions in three patients and their performance on standardised psychometric tests. The authors suggest that the general transcription factors *GTF2I* and *GTF2IRD1*, at the telomeric end of the WSCR, are likely to play a disproportionate and crucial role in the development of neural pathways involved in visuospatial cognition. Dai *et al*. [26] also sought to delineate the role of these transcription factors in the WS phenotype, finding that an individual with an atypical deletion that included *GTF2IRD1*, but not *GTF2I*, presented with poor performance on a number of spatial subtests from the Wechsler Preschool and Primary Scale of Intelligence- Revised (WPPSI-R) [27], including ‘Block Design’, ‘Object Assembly’, and ‘Mazes’. Preservation of the normal copy number of *GTF2I* in this individual was argued to contribute to the relative strengths found in those non-verbal cognitive measures that did not require visual-motor integration (‘Picture Completion’ and ‘Geometric Design Recognition’), and in verbal cognition. It appears, then, that the general transcription factor genes at the telomeric end of the WSCR are likely to make a significant contribution to the WS visuospatial phenotype.

The general transcription factor (GTF) genes are also thought to have widespread effects on the expression of other genes [28], and are differentially expressed in the developing brain compared to the adult brain [1]. The impact of mutations of these telomeric genes, in particular on the expression of other genes, may therefore be diverse and have varying cascading effects throughout development. As such, to gain a more thorough understanding of the role, combinatorial effects, and penetrance of all 28 transcripts within the WSCR (particularly the GTF genes) on the phenotypic profile of WS, research must examine the different effects of specific genetic mutations across partial deletion patients with differing genomic makeup.

It is not only at the level of the genotype that more in-depth research has been necessary; the phenotypic outcome also calls for more subtle analyses rather than solely relying on psychometric spatial tasks. Indeed, recent research has sought to elucidate whether there are dissociable deficits within the visuospatial domain in individuals with the full WS deletion. In particular, the different cognitive demands associated with understanding the location of the self (‘egocentric’ spatial representations) and object-based spatial relationships (‘allocentric’ spatial representations) have been examined through the use of both small-scale table-top tasks [for example, [20] and large-scale navigation tasks [for examples, [14,23]. The findings of such studies have identified difficulties in the use of both egocentric and allocentric spatial representations in WS. However, little can as yet be concluded regarding the genetic contributions to the specific deficits in the use of these different spatial frames of reference. Given the importance of these aspects of spatial representation to human navigational abilities, and hence to every-day living, attempts to examine the genotypic correlations with these specific spatial deficits must address the use of these different cognitive processes both in individuals with the full WS deletion and in individuals with partial deletions within the WSCR. We therefore argue that it is critical to test, not only performance on psychometric spatial tasks, but particularly performance on novel, hypothesis-driven small-scale tasks and navigational large-scale search tasks that tap into egocentric and allocentric spatial cognitive demands.

Here, we present case studies of spatial cognition in two individuals (HR and JB) neither of whom meets both genetic and phenotypic criteria for a typical diagnosis of WS, but present with contrasting partial genetic deletions within the WSCR. Previous comparisons of the socio-cognitive profiles of these two patients highlighted the different levels of social impairment that result from such contrasting deletions [12]. The current study focussed on visuospatial cognition and examined the impact of these differing genetic deletions in the WSCR by investigating performance of HR and JB, using a range of table-top psychometric tasks, and small- and large-scale spatial tasks. Visuospatial abilities of both individuals were also compared to performance on the same tasks previously reported both in typical development and in individuals with the full WS genotype.

## Methods

### Participants

**Figure 1:**
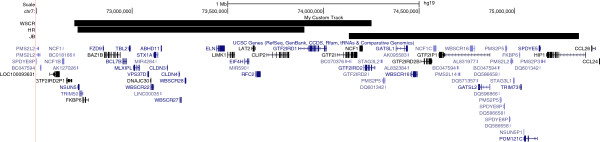
Summary of the genomic co-ordinates and genes located in the typical approximate 1.55 Mb deletion in Williams Syndrome and the individual deletions of HR and JB (build hg19).

Data are presented from two individual cases with different genetic deletions occurring within or overlapping the WSCR. The extent of genetic deletion in each individual was determined using array comparative genome hybridisation (aCGH). HR is a female, aged 11 years 9 months and has a deletion of approximately 1 Mb which spans from the centromeric end of the WSCR at *NSUN5* to *GTF2IRD1* (72716513 to 73900000). The breakpoint lies within *GTF2IRD1* such that HR is partially deleted for *GTF2IRD1*, leading to reduced expression and thus haploinsufficiency for this general transcription factor [29]. The remaining three telomeric genes of the WSCR (*GTF2I*, *NCF1*, *GTF2IRD2*) are present in HR. JB is a male, aged 14 years 2 months and, in contrast with HR, has a deletion of approximately 2 Mb with a breakpoint in the distal WS region within (and so partially deleted for) *GTF2I* that extends beyond the WSCR in the telomeric direction (including a deletion of *NCF1* and *GTF2IRD2* within the WSCR) to *HIP1* (74133268 to 75333536). JB’s deletion therefore contains 21 reviewed RefSeq genes based on the most recent build of the human genome (hg19). Figure 1 shows the genetic deletions of HR and JB together with the regions typically deleted in patients with WS.

Both participants underwent a broad range of cognitive-functioning and visuospatial tasks. Performance by HR and JB on a mental rotation task, a visual perspective-taking task, and a large-scale navigation strategies task is compared to performance by typically developing (TD) individuals aged five, six, eight and ten years, and a group of individuals with full WS, previously reported by Broadbent *et al*. [21,23] (see Tables 1 and 2 for participant details). Furthermore, performance by HR and JB on a large-scale route learning task is compared to performance from two groups of TD individuals aged six to seven years and eight to nine years and a group of individuals with full WS, previously reported by Farran and colleagues [30] (see Table 3). The TD group age ranges were selected as marked developmental changes in the spatial abilities measured here occur during this period [31-35]. In addition, the range of verbal and non-verbal abilities in individuals with WS corresponds with performance on the same tasks in TD children within these chronological age ranges [21,23,30]. Ethical approval for the study was obtained from the Institute of Education ethics committee.

**Table 1:** Mental rotation and visual perspective-taking scores for HR and JB compared to WS and TD group means (SD)

	**HR**	**JB**	**WS (N = 20)**	**TD 5 years (N = 16)**	**TD 6 years (N = 16)**	**TD 8 years (N = 17)**	**TD 10 years (N = 16)**
Chronological age (years; months)	11;09	14;02	24;05 (10;07)	5;06 (0;04)	6;08 (0;03)	8;03 (0;05)	10;01 (0;04)
Mental rotation (% correct rotation trials)	64.29	89.29	52.86 (17.48)	69.19 (24.11)	70.54 (21.61)	87.61 (8.29)	89.73 (10.90)
Perspective-taking (% correct L/R rotation trials)	100	100	32.13 (14.93)	46.88 (32.57)	60.92 (30.52)	91.27 (12.14)	91.96 (13.77)

**Table 2:** Navigation strategies scores for HR and JB compared to TD and WS group means (SD)

	**HR**	**JB**	**WS (N = 17)**	**TD 5 years (N = 16)**	**TD 6 years (N = 15)**	**TD 8 years (N = 17)**	**TD 10 years (N = 16)**
Chronological age (years; months)	11;09	14;02	21;10 (8;06)	5;07 (0;04)	6;08 (0;03)	8;03 (0;05)	10;01 (0;04)
Navigation strategies (trials to learn)	4	6	4.76 (2.08)	4.38 (1.71)	4.64 (1.99)	3.76 (.97)	2.75 (.68)
Navigation strategies (allocentric score)	10	4	2.94 (1.43)	3.00 (3.85)	3.00 (2.29)	4.65 (2.74)	6.31 (3.79)

**Table 3:** Route learning scores for HR and JB compared to WS and TD group means (SD)

		**HR**	**JB**	**WS (N = 19)**	**TD 6 to 7 years (N = 20)**	**TD 8 to 9 years (N = 20)**
Chronological age (years; months)		11;09	14;02	22;04 (9;00)	6;10 (0;09)	8;10 (0;08)
Route learning (trials to learn)		3	10	6.26 (2.26)	6.50 (2.26)	4.40 (1.60)
Route learning (errors during learning trials*)*		1	15	8.47 (7.03)	7.35 (4.17)	3.40 (2.78)
Route learning (recall of landmarks)	Junction (maximum: 8)	8	2	5.45 (3.15)	5.25 (1.97)	5.05 (2.33)
	Path (maximum: 8)	7	1	2.90 (2.02)	3.45 (1.67)	4.05 (1.47)

### Psychometric cognitive functioning measures

‘Verbal’, ‘Non-verbal Reasoning’, and ‘Spatial’ intellect were assessed using the British Ability Scales-II School Age (BAS-II) [36]. Verbal scores from the BAS-II are calculated from the Verbal Similarities and Word Definitions core scales, and Non-verbal reasoning is calculated from the Matrices and Qualitative Reasoning core scales. Spatial scores are derived from the Recall of Designs and Pattern Construction core scales. The Raven’s Coloured Progressive Matrices (RCPM) [37] was included as an additional measure of non-verbal ability as it has been established as a sensitive and reliable measure of non-verbal functioning in WS [38].

### Experimental spatial measures

Two small-scale and two large-scale spatial tasks were chosen in order to measure a diverse range of visuospatial abilities in HR and JB, which have been previously used to highlight specific spatial deficits and atypical functioning in individuals with WS. A behavioural distinction can be made between the mental transformation of objects and imagined self-rotations (perspective-taking) [33,39], with performance on these two types of task associated with activity in dissociable, although overlapping, neural systems [for example, [40]. Tasks requiring the use of different types of mental transformation (namely, object-based mental rotation and visual perspective-taking), measures of egocentric and allocentric spatial-coding and route learning in large-scale space were therefore conducted.

#### *i) Mental rotation (MR) task*

The ability to imagine the rotation of a 2D object at varying degrees of displacement from upright was measured using the monkey MR task, based on the classic mental rotation paradigm by Shepard and Metzler [41]. Participants were asked to view images of two monkeys above a horizontal line and one monkey below the line at varying degrees of rotation from upright, presented on a 14” laptop computer screen. Participants had to select which of the two monkeys on the top matched the rotated one underneath, indicating their response by pressing a left or right button on a keyboard in front of them. The MR task consisted of 28 rotation trials, and four control trials where the monkey underneath was not rotated (0° position). The test positions of the target monkey included 45°, 90°, 135° and 180° clockwise rotations and -45°, -90°, -135° anticlockwise rotations [for full details of task design, see [21].

#### *ii) Visual perspective-taking (VPT) task*

The VPT task was used to examine participants’ ability to imagine the self rotating around a circular array of four objects, and adapted from VPT tasks used in previous studies [35,39]. Throughout the task, the participant was asked to imagine looking at the array from different viewpoints as well as a series of questions about the position of the different objects from the imagined perspectives (which object would be to your left, right, furthest, nearest). Given that young children and some individuals with WS have difficulties distinguishing their left from right sides [42], each participant was given a sticker on one hand (randomised left and right across participants) so that instead of declaring a left or right turn, they stated whether they would turn to their ‘sticker’ or ‘no-sticker’ side. This was similar to previously reported methods [35] that significantly improved performance on such tasks. Using two separate arrays, each participant was tested on a variety of imagined displacements of the self (45°, 90°, 135° and 180°, collapsed across clockwise and anti-clockwise rotations). The test consisted of 28 rotation trials and four control trials where no imagined rotation was required [for full details of task design, see [21].

Although validity and reliability of the experimental measures used in this study have not been established, on account of their novelty, robust levels of convergent validity and reliability of the measures on which the VPT task is based have been reported [39]. In addition, it has been established that VPT measures are dissociable from mental rotation tasks [39], providing support for discriminant validity of the small-scale experimental measures used here.

#### *iii) Large-scale navigation strategies task*

When navigating through a virtual environment (VE) compared to a real one, behavioural performance is largely equivalent [43], and broadly comparable cognitive mechanisms underlie learning to orientate [44]. However, despite showing substantial shared neural activation, there are some differences in neural activity for virtual versus real-world environments [43]. This has been attributed to the finger and hand movements required to navigate VEs [43].

An interactive VE maze was presented to participants to examine the spontaneous strategy used by each participant during large-scale navigation, and also whether the participant could develop an understanding of the spatial relationships between landmarks in the environment (an allocentric understanding of space), when required. The VE consisted of a brick wall cross-maze with four paths radiating from a central square and six distal landmarks surrounding the perimeter; a modified version of the ‘starmaze’ paradigm used in other navigation studies [for examples, [45,46]. The VE presented participants with an environmental layout within which individuals could use either a sequential egocentric strategy (using the same sequence of left-right body turns) or an allocentric strategy to navigate, or a combination of the two (mixed strategy) for [full details of maze design, see [23].

The participant was first shown the correct route through the VE from start to finish, by being asked to follow a green grass path. They then navigated the route without the aid of the grass path by using the arrow keys on the keyboard, referred to as learning trials. Learning trials were repeated until the participant had completed the route without errors on two trials. Participants were then asked to continue finding the exit on 12 further trials, four of which were ‘spontaneous strategy trials’ where the participant was started unknowingly from a different starting position in the environment.

After the spontaneous strategy trials, participants were taught a new route in the environment. Following successful learning, they were then asked to find the new exit six times, using the quickest route, when this time they were knowingly started from different starting places (a test of allocentric knowledge). An ‘allocentric score’ was calculated from these six trials, with two points given for each allocentric strategy used, one point for a mixed strategy, and zero points for an egocentric strategy or incorrect trial (maximum twelve points). As a further measure of their mental representation of the spatial relations of the environmental layout, at the end of the task participants were shown a selection of six birds-eye-view maps and asked to choose the correct layout of the environment through which they had been navigating.

#### *iv) Large-scale route learning task*

Participants viewed a second VE task presented on a 17-inch laptop screen. The VE consisted of a brick wall maze with six junctions and sixteen landmarks. Landmarks were either close to junctions (junction landmarks), or were mid-way along a path section (path landmarks) [for full details of maze design, see [30].

The experimenter showed each participant the correct route through the maze from start to finish. The participants then navigated the route themselves, using the arrow keys on the keyboard, referred to as learning trials. Learning trials were repeated until the participant had completed the route without errors on two consecutive trials. Participants were then tested on their recall of the landmarks. The number of learning trials required and the number of cumulative errors made across learning trials provided a measure of route learning. The number of landmarks recalled provided a measure of whether landmarks were used (a key component of route learning in the typical population).

### Statistical analyses

Comparisons of performance on MR, VPT and large-scale navigation and route learning tasks by HR and JB with WS and TD data were conducted using Crawford-Howell modified *t*-tests for case–control comparisons [47], developed to compare an individual's (N = 1) score to that of a small control group or normative sample (where N < 50). The program ‘Singlims_ES.exe’, was used to analyse the data [48], available at: http://homepages.abdn.ac.uk/j.crawford/pages/dept/psychom.htm [last accessed 3 May 2014]. The modification of the independent samples *t*-test takes into account the mean and standard deviation for the comparison group on the task (and group N), and the raw score of the single case. A point estimate of the effect size for the difference between the case and control (z-cc) with an accompanying 95% CI are also reported for statistically significant findings.

## Results

### Psychometric cognitive functioning

HR had a Verbal score of 80 (ninth percentile) and Spatial score of 73 (fourth percentile) on the BAS-II, demonstrating relatively impaired performance in both of these domains. In contrast, she presented with a relative strength in Non-Verbal Reasoning, with a score of 98 (45^th^ percentile). HR’s age-appropriate score of 32 on the RCPM also indicated a cognitive strength.

In contrast to HR, JB showed impaired performance on all measures of the BAS-II, with a Verbal score of 59 (0.3 percentile), a Spatial score of 47 (0.1 percentile), and a Non-Verbal Reasoning score of 65 (first percentile). Similarly, JB presented with impaired performance on the RCPM, with a score of 18, an age-equivalent level of 7 years pointing to a score of only half his chronological age.

### Experimental spatial measures

Descriptive statistics for HR, JB, TD and WS groups from the mental rotation and visual perspective-taking tasks are shown in Table 1, navigational strategies tasks are shown in Table 2, and route learning task are shown in Table 3.

#### *i) Mental rotation (MR) task*

On the monkey mental rotation task, HR scored 18 out of 28 on rotation trials (64.29% correct). This was in line with the level of performance observed in TD 5 and 6 year-olds (*t* = -.19, *p = .42;* and *t* = -.28, *p = .39,* respectively), but reliably poorer than performance seen in TD 8 and 10 year-olds (t = -2.61, *p = .01*, z-cc = -2.68 (CI = -3.71 to -1.64), and *t* = -2.26, *p = .02*, z-cc = -2.33 (CI = -3.29 to -1.36), respectively). HR’s performance was at a similar level to that of individuals with WS (*t* = .64, *p = .27*), who were at chance level. Conversely, JB scored 25 out of 28 on rotation condition trials (89.29% correct), demonstrating a high, near-ceiling level of performance on this task. Unlike HR, JB scored significantly above the WS group on this task, *t* = 2.03, *p = .03*, z-cc = 2.08 (CI = 1.29 to 2.87). Although JB’s performance was at a similar level to TD 8 and 10 year-olds, who perform at near-ceiling on this task, using the statistical methods noted above, scores were not reliably different from any TD groups (5 years: *t* = .81, *p = .22;* 6 years: *t* = .84, *p = .21*; 8 years: *t* = .19, *p = .42*; 10 years: *t* = -.04, *p = .48*). That said, given JB’s high level of performance compared to individuals with WS, clearly, performance on this task is a key indicator of genotype-phenotype relations for the genes under investigation here.

#### *ii) Visual perspective-taking (VPT) task*

HR showed no deficits on this task, performing at ceiling with all 28 self-rotation responses correct, HR’s score for left-right responses on the task being 100% (14/14). This reliably exceeds performance by individuals with WS (*t* = 4.44, *p < .001*, z-cc = 4.55, (CI = 3.04 to 6.04)), who show substantial deficits on self-rotations greater than 45° from their own vantage point. Similarly, JB showed no deficits on this task, apart from on trials when asked to state the ‘nearest’ object. This may have reflected a difficulty in understanding the meaning of the word, although he did get this correct on the control trial. On the trials requiring a left-right response, JB performed at ceiling with all 14 self-rotation responses correct. Again, this performance was greater than that observed in individuals with WS (*t* = 4.44, *p < .001*, z-cc = 4.55, (CI = 3.04 to 6.04)), and at a similar level to TD 8 and 10 year-olds (*t* = .69, *p = .25*; and *t* = .57, *p = .29*, respectively), many of whom also performed at ceiling. Of note however, is that neither HR nor JB performed reliably above TD 5 and 6 year-olds (*p > .05* for all), given that some individuals in these groups also performed well on this task. This may be a reflection of the stringent nature of the modified *t*-test, given that age-related differences were found between TD groups on this task [21].

In sum, HR and JB had similar performance on this task, demonstrating no deficit in VPT. Given ceiling performance, this task may not have been sensitive enough to reveal differences between HR, JB and older TD children. However, performances by both PD patients were indicative of a visuospatial profile different to that typically seen in individuals with WS. This high level of performance by JB was particularly surprising, given his poor scores on psychometric measures of cognitive functioning, although it is in line with his good mental rotation performance. The high level of performance in JB on these two experimental tasks provides a vital clue to the role(s) of the GTF genes within the WSCR.

#### *iii) Large-scale navigation strategies task*

HR learnt the route quickly, in only four trials. Although this was marginally slower than the ceiling performance seen in TD 10 year-olds, *t* = 1.78, *p = .05,* z-cc = 1.84 (CI = 1.01 to 2.64), this was in line with TD children aged 8 years and younger and individuals with WS, who all reached criteria after a short number of trials (*p > .05* for all). On trials examining the spontaneous navigation strategy, HR did not use a consistent strategy, but was incorrect on the first trial, then used an egocentric strategy for one trial and a mixed strategy on the final two trials. Comparable with spontaneous performance in typical adults [46], TD children predominantly rely on the use of a sequential egocentric strategy to navigate on this type of task [23,45], a strategy associated with preferential activation in the dorsal striatum and left hippocampus [49]. Individuals with WS, however, tend to rely on a mixed strategy on this task, likely due to the use of visual-matching and a reliance on landmarks for guidance. In contrast to individuals with WS, on trials examining the ability to use an allocentric strategy when prompted, HR was able to use this effectively, showing an allocentric strategy and ability to take the shortest route on 5/6 trials. The calculated allocentric score for HR on this task was at an age-appropriate level, in line with TD 10 year-olds (*t =* .95, *p = .18*), and at a level significantly higher than TD 5 year-olds (*t* = 1.76, *p = .*04, z-cc = 1.82 (CI = .99 to 2.62)); 6 year-olds (t = 2.96, *p = .01* z-cc = 3.06 (CI = 1.82 to 4.28)); and 8 year-olds (*t* = 1.89, *p = .04*, z-cc = 1.95 (CI = 1.12 to 2.76)). Similarly, HR scored significantly higher than individuals with WS (*t* = 4.79, *p < .001*, z-cc = 4.94 (CI = 3.17 to 6.69)), who demonstrate particular deficits in the use of spatial relational or allocentric frames of reference for navigation [23]. HR also chose the correct map layout, further suggesting an appropriate spatial relational representation of the environmental layout.

JB took a significantly greater number of trials than TD 8 and 10 year-olds to learn the route (*t* = 2.24, *p = .02* z-cc = 2.31 (CI = 1.38 to 3.22), and *t* = 4.64, *p < .001,* z-cc = 4.78 (CI = 3.01 to 6.53), respectively), reaching criteria only after 6 trials. JB’s performance was not reliably different to that observed in TD 5 year-olds (*t =* .92, *p = .19*), 6 year-olds (*t =* .66, *p = .26*), or WS (*t =* .58, *p = .29*). On trials examining the spontaneous navigation strategy, JB made errors on two trials, but used an egocentric strategy on the other two trials, in line with the strategy predominantly observed in TD children. On trials examining the ability to use an allocentric strategy when required, JB made errors on half (3/6) of the trials. However, on the other trials, JB demonstrated an ability to use view-matching to search for the correct path, albeit in an inefficient and laborious manner that did not include taking the shortest route (2 trials as mixed strategy, 1 as allocentric). Although this resulted in an allocentric score that could be considered delayed when contrasted with TD 10 year-olds, analyses did not yield any significant differences between JB and any TD group or the WS group, (*p > .05* for all).

JB also correctly chose the environmental layout from the map selection. This suggests that JB may have developed a partial mental representation of the environmental layout. It should be noted, however, that a few WS participants also chose the correct layout despite showing no other use of an allocentric strategy for navigation. Although it cannot be concluded that JB developed an allocentric spatial representation of the environment to aid navigation, JB’s large-scale spatial performance appeared stronger than typically observed in WS, and particularly unexpected given JB’s very low scores on measures of non-verbal reasoning and psychometric measures of spatial intellect.

#### *iv) Large-scale route learning task*

Similar to her performance on the navigation strategies task, HR performed at a high level on the large-scale route learning task, in line with TD 8 to 9 year-olds on the number of trials taken to learn the route, (*t = -.*85, *p = .20*). She learnt the routes very quickly, in three learning trials, although performance was only marginally superior to that of the WS group and TD 6-7 year-olds (*t =* -1.41, *p = .08*, z-cc = -1.44 (CI = -2.08 to -.78) and *t* = -1.51, *p = .07*, z-cc = -1.55 (CI = -2.19 to -.88), respectively). HR also produced only one error on learning trials. This was, again, in line with the oldest TD group of 8 to 9 year-olds (*t = -.*84, *P = .20*), and so could be indicative of age-appropriate performance, although was only marginally superior to TD 6 to 7 year-olds (*t = -*1.49, *p = .08*, z-cc = -1.52 (CI = -2.16 to -.86)) and not significantly different from the WS group (*t = -*1.04, *p = .16*), given that some individuals in these groups also performed well on this task. HR was able to recall the location and identity of landmarks on all eight trials for junction landmarks, and on seven out of eight trials for path landmarks. Given that memory for the location and identity of *junction* landmarks was also at a high level in TD and WS groups, HR did not differ significantly from performance in these groups (TD 6 to 7 years: *t* = 1.36, *p = .09*; TD 8 to 9 years: *t =* 1.24, *p = .12*, and WS: *t =* .79, *p = .22*). HR’s memory for the location and identity of *path* landmarks however was significantly stronger than the WS group (*t =* 1.98*, p = .03*, z-cc = 2.03 (CI = 1.22 to 2.82)), and both TD control groups (TD 6 to 7 years: *t =* 2.08, *P = .03*, z-cc = 2.13 (CI = 1.32 to 2.92) and TD 8 to 9 years: *t =* 1.96, *p = .03*, z-cc = 2.01 (CI = 1.23 to 2.77)). HR did not show the advantage for junction over path landmarks observed in WS and TD. This could be evidence for a somewhat atypical strategy, but it most likely reflects ceiling performance.

Like his performance on the navigation strategies task, JB took a large number of trials to learn the route in this task (ten learning trials) and made fifteen errors, mainly due to the perseveration of errors at the same junctions over consecutive learning trials. Compared to individuals with WS, JB required marginally more trials to learn the route (*t =* 1.61, *p = .06*, z-cc = 1.66 (CI = .95 to 2.35)), but did not make significantly more errors (*t =* .91, *p = .19*), also due to a large number of perseverative errors in the WS group [30]. Compared to TD groups, however, JB required marginally more trials and made significantly more errors than 6 to 7 year-olds (*t =* 1.51, *p = .07*, z-cc = 1.55 (CI = .88 to 2.19) and *t =* 1.79, *p = .04*, z-cc = 1.84 (CI = 1.09 to 2.55), respectively), and required significantly more trials and made more errors than 8 to 9 year-olds (*t =* 3.42, *p = .001*, z-cc = 3.50 (CI = 2.31 to 4.68) and *t =* 4.07, *p < .001*, z-cc = 4.17 (CI = 2.78 to 5.55), respectively).

JB’s memory for the location and identity of the landmarks was impaired, with a score of two out of eight and one out of eight for recall of junction and path landmarks respectively. However, JB’s memory for junction landmarks was not found to be poorer than in WS (*t =* -1.07, *p = .15*), was only marginally poorer than TD 6 to 7 year-olds (*t =* -1.61, *p = .06*, z-cc = -1.65 (CI = -2.32 to -.96)) and not different from TD 8 to 9 year-olds (*t =* -1.28, *p = .11*). For path landmarks, JB scored in line with the WS group (*t =* -.92, *p = .19*) and TD 6 to 7 years (*t* = -1.43, *p = .08*) but significantly below TD 8 to 9 years: *t =* -2.03, *p = .03*, z-cc = -2.08 (CI = -2.85 to -1.28). JB also did not show an advantage of junction over path landmarks as observed in WS and TD, but this might simply reflect his low scores, which were almost at floor.

## Discussion

The uneven cognitive profile in WS has provided insights, but also controversies, into the genetic contributions to human visuospatial cognition. Indeed, the elucidation of which of the 28 WSCR genes play a role in the visuospatial phenotype in WS is complex. Initial studies had implicated *LIMK1* as a major contributor to the visuospatial deficits in WS, on the basis of human partial deletion patients and mouse models [13,24]. However, subsequent work on other partial deletion patients showed that if *LIMK1* played a role, it had to be in combination with other genes at the telomeric end of the WSCR [9,14,15]. The two genetically contrasting case studies presented in this paper provide further insight into the possible combinatorial effects of genes within the WSCR, including the role of the general transcription factors on *some* aspects of visuospatial cognition. Moreover, whereas previous studies had compared small-scale, table-top spatial deficits in WS with large-scale navigational deficits in the mouse, which place very different cognitive demands on each species, the current study examined both small- and large-scale visuospatial abilities in the same participants. This more in-depth analysis of visuospatial cognition is critical if we are to understand genotype/phenotype relations in Williams syndrome.

An interesting pattern of strengths and weaknesses within the spatio-cognitive domain emerged in both participants. In particular, HR showed poor performance on psychometric measures of spatial intellect (BAS-II) and mental-rotation, alongside a relative strength in non-verbal reasoning and VPT. HR’s performance on our battery of tasks was, despite her deletion of over 24 genes in the WSCR, therefore not reflective of the cognitive profile of individuals with the full WS deletion. Indeed, HR performed at a level significantly above that observed in individuals with full WS on the VPT task, but below an age-appropriate level, and in line with individuals with WS on the MR task.

Neither was the pattern of performance across the small- and large-scale experimental tasks in HR entirely typical. Relatively poor performance by HR both on the psychometric measure of spatial intellect and the mental rotation task, alongside proficient performance on VPT, large-scale navigation and route learning, reflects the multi-faceted nature of spatial cognition. In typical individuals, moderate correlations are found between performance on psychometric and small-scale spatial measures and large-scale spatial abilities [50,51]. However, in typical development, although performance on tests of visuospatial memory correlates highly with route learning ability, this is largely moderated by executive control [52]. As such, although somewhat overlapping, small and large-scale spatial abilities are partly dissociable and it can be inferred that visuospatial abilities may be differentially affected by divergent genetic deletions.

What about the relationship between VPT and navigation strategies? Moderate correlations are also found in typical adults between VPT and the ability to use an allocentric navigation strategy in large-scale space [53]. Accordingly, HR’s high level of performance on the VPT and navigation tasks (in contrast to individuals with the full WS deletion, who show substantial deficits on both of these tasks) suggests that she may be able to use this ability to spatially update the location of the self and to navigate successfully following a change in position in a large-scale familiar environment. That said, little is known about the extent to which performance on VPT tasks account for the variance in navigational abilities across development. Indeed, a high level of performance in JB on the VPT task contrasted with his poor navigational ability and low overall cognitive functioning (discussed later) suggests that they are not wholly associated, and may be differentially affected by genotypic variation.

In summary, in spite of HR’s deletion of over 24 genes on the WSCR, including haploinsufficiency for *GTF2IRD1*, only some atypical spatio-cognitive functioning was observed that resembles that of individuals with full WS. These findings suggest that the retention of the more telomeric 7q11.23 genes contribute to the relatively good large-scale visuospatial performance observed in HR, particularly in the face of her relatively low level of cognitive functioning as measured on Verbal and Spatial psychometric scales. However, it remains inconclusive from this whether each of these telomeric genes - *GTF2I*, *NCF1* and *GTF2IRD2*- play an equal role in large-scale spatial cognition.

If *LIMK1* (deleted in HR) plays a role in spatial cognition, then it may be that the preservation of the most telomeric genes within the WSCR allowed for the development of compensatory spatial strategies in HR, reflected in part by her good performance, albeit with some atypical features, on the large-scale navigation and route learning tasks. That said, it is difficult to disentangle between whether such compensation is due to a genetic mechanism, or to the application of an alternative strategy that transpired through specific training. Further consideration of the modulatory role of both *LIMK1* and GTFs on various visual processes within the WS phenotype, particularly in regards to their expression in different neural tissues [for example, [54], is therefore imperative. As such, more comprehensive phenotypic studies at different levels of the nervous system would highlight more specifically the profile of visuospatial strengths and weaknesses in relation to the genetic underpinnings.

The profile of HR was presented alongside that of JB, an individual with a contrasting hemizygous deletion, which extends telomerically from within *GTF2I* to beyond the WSCR. Two quite differing cognitive profiles emerged from HR and JB. At the cognitive level, JB presented with profound impairments across the Verbal, Non-verbal and Spatial domains, as measured using the BAS-II. Despite his preservation of the majority of the genes on the WSCR, this profile of deficits is expected, given the probable role of the telomeric genes on the expression of other genes [1] as well as the role of *GTF2I* on general intellectual ability [10]. It should be noted however, that JB’s deletion includes haploinsufficiency for up to 21 genes, many of which have unknown function. As such, it remains unclear what contribution they make to his profile. For example, haploinsufficiency for *HIP1* (deleted in JB) has been reported to be associated with neurological and neuropsychological deficits including epilepsy and autistic traits in other individuals with atypical deletions flanking the WSCR [55]. It is therefore important to take into account that JB has a large number of genes deleted outside of the WSCR, and is likely to have altered expression of *GTF2I* and is haploinsufficient for *HIP1*, deletions of which are known to contribute to lower cognitive functioning. Conclusions regarding comparisons of the two cases are therefore tentative. That said, the inclusion of these two cases together provides insight into the differential effects of deleted genes within 7q11.23 on visuospatial abilities at different spatial scales, in the face of differing overall levels of intellectual ability.

Surprisingly, JB performed at a very high level on the small-scale mental rotation and VPT tasks, with scores significantly above those observed in individuals with full WS. By contrast, on both of the large-scale navigation tasks, and like individuals with WS, JB took a long time to learn the route, with performance below TD 8 and 10 year-olds. Allocentric spatial coding was also somewhat compromised, although not significantly different from any TD or WS groups (possibly due to the stringent nature of the modified *t*-test, given that differences are previously reported across TD and WS groups on allocentric score [23]). Despite these impairments on large-scale spatial tasks, his performance reflected an overall profile unlike that observed in individuals with the full WS deletion. Indeed, the uneven profile of JB’s relative strengths and weaknesses across different spatial scales in the visuospatial domain was also not comparable to that observed in TD individuals. Furthermore, given that JB performed at a high level on mental rotation and VPT tasks, his relatively poorer performance on large-scale navigation cannot purely be considered a reflection of low general cognitive ability. Instead, these findings strongly indicate the role of other genes at 7q11.23 in mental rotation ability other than *GTF2I* or *GTF2IRD2* (for which JB is haploinsufficient). Similar robust conclusions cannot be made regarding the role of WSCR genes on VPT ability, given that both JB and HR performed at a high level on this task, and the vastly differing intellectual profiles of the two cases. However, there may be combinatorial effects of the GTFs and other more centromeric WSCR genes on VPT, although this can only be tentatively inferred given the difficulties in drawing direct comparisons from the two cases presented here.

Across the tasks in the current study, neither HR nor JB presented with a clear WS spatio-cognitive profile, and both performed outside of the typically-observed variations in performance by individuals with full WS. A spatial advantage, particularly for mental rotation [56], is usually attributed to males, which could, at first blush, be considered to explain the higher level of performance on this task by JB than HR. However, no gender differences were apparent in the TD or WS participants across the battery of spatial tasks employed here, including in mental rotation, suggesting that differences in the two cases presented here are not likely related to gender. Furthermore, on other tasks HR typically performed at a higher level than JB, which is more likely a reflection of their differences in overall intellectual functioning, than of gender.

These contrasting profiles pose an interesting question as to the combinatorial effects of genes at locus 7q11.23 on the WS visuospatial phenotype, particularly those at the telomeric end. As mentioned, HR’s deletion includes that of *LIMK1*, a gene that had gained much attention in the search for mapping from genotype to spatial phenotype. Although *limk1* plays a critical role in long-term potentiation in the mouse hippocampus [24], research in humans with atypical deletions in the WSCR has challenged the independent contribution of *LIMK1* to the visuospatial deficits seen in WS [for examples, 9,14,15]. More recently, the chromosomal region telomeric to RFC2, including *CYLN2*, *GTF2IRD1*, and *GTF2I*, has become a focus of interest as a possible contributor to the spatial cognitive profile in WS [6,57]. *CYLN2* (also known as *CLIP2*), for example, encodes CLIP-115, which is expressed in dendrites and cell bodies in a number of brain regions, and has been found to effect hippocampal memory processes [57]. HR is not deleted for *GTF2I* or other more telomeric genes, but her deletion does include a reduced expression of (and thus haploinsufficiency for) *GTF2IRD1*. Given HR’s difficulties in mentally rotating objects, these results support previous findings that haploinsufficiency for *GTF2IRD1* in combination with other 7q11.23 genes such as *CYLN2* or *LIMK1* may play a role in some of the (small-scale) visuospatial cognitive deficits observed in individuals with WS [14,58]. This is supported by JB who is not deleted for either of these genes and performed well on the mental rotation task. Nonetheless, we cannot rule out that the deletion of the other more telomeric general transcription factors has impacted the expression of *CYLN2* and other 7q11.23 genes in JB [for example, see [58]. It also remains unclear whether the deletion of other genes beyond the WSCR plays a role in the expression of intact genes within the WSCR.

Initially, the examination of visuospatial performance by HR and JB seems indicative of the additive effect of deleting each of the *GTF2I* family genes on the severity of cognitive impairment, and is in line with other findings in individuals with extended deletions (approximately 1.8 Mb) that encompass *GTF2IRD2*, who present with significantly greater neurological impairments than individuals with shorter deletions typical of WS [59]. However, although we cannot dismiss the effects of other deleted genes in JB that extend beyond the WSCR on cognitive functioning, JB’s high level of performance on small-scale mental rotation and VPT rules out the conclusion of a general deficit, and excludes the role of genes telomeric to *GTF2I* in these specific small-scale spatial abilities. Indeed, the preservation of most other genes within the WSCR in JB suggest that spatial skills may be differentially affected when GTF genes are deleted in combination with other more centromeric genes on the WSCR. Of note here, is that information regarding whether the deleted region in any participants with WS in the study also included *NCF1* and *GTF2IRD2* (an approximate 1.8 Mb deletion that occurs in around 5% of cases) was not obtained. This is because, for the majority of individuals with WS, the genetic contributor to diagnosis is via a Fluorescent *in situ* Hybridisation (FISH) test, which does not provide deletion size information. We assume that the majority of our sample had a standard 1.55 Mb deletion in line with 95% of the WS population. Inclusion of individuals with greater deletions would have resulted in an underestimation of the ability of the WS group. That said, JB’s deletion did include these genes and was found to perform at a higher level than WS on some tasks and in line with WS on others, findings that would not have transpired had WS group data been compromised due to the inclusion of such individuals.

While the current study has highlighted candidate genotype/phenotype relations in the presentation of two contrasting case studies, it is clear that future studies need more in-depth genetics and phenotypics, measured over developmental time [60]. Indeed, with reference to genetics, it would be preferable to perform DNA and RNA sequencing to delineate the precise genomic boundaries of the deleted regions, and to examine gene expression patterns in the WSCR region and throughout the genome [4]. However, it is also critical that such detailed genetic studies be accompanied by in-depth phenotypic studies, particularly at the cognitive level. The phenotypic analysis requires a broad spectrum of tasks, both psychometric and hypothesis-driven, like in the current study, [see also, [15] as examining performance only on psychometric tasks [for example., [4] does not yield a detailed account of phenotypic expression, particularly regarding the multi-faceted nature of spatial cognition. The fact that genome function is modified over developmental time as a function of the epigenome requires a longitudinal assessment from infancy onwards of the details of the changing phenotype. Furthermore, case studies do not make it possible to ascertain whether gender influences gene expression in the WSCR nor whether, in our particular cases, JB or HR have other genetic mutations elsewhere in their genome, outside the WSCR, that may affect general intellectual outcome.

Genetic and phenotypic examination of other family members would be critical to complete the picture of such case studies. Thus, numerous factors that include changes in gene expression across development, but also environmental influences, education and other individual differences may contribute to the complex phenotypic outcomes in HR and JB. For this reason, conclusions regarding genotype-phenotype associations from individuals with partial deletions must always be considered with some caution and supplemented by appropriate animal models. Moreover, such genotype-phenotype correlations should take into account the possible role of candidate genes in the development of other neural tissues. For example, Castelo-Branco and colleagues [54] examined the contribution of the general transcription factors to the neural retinal phenotype in WS, finding patterns of visual impairment that were separate from the known cortical dorsal-stream phenotype. This highlights the important nature of in-depth phenotypic analyses in order to draw more robust conclusions as to the contributions of specific genes to cognitive phenotypes.

## Conclusions

The examination presented in this paper of two individuals with such contrasting genetic deletions within the WSCR provides further understanding as to candidate genotype relations to the WS visuospatial cognitive phenotype. The pattern of strengths and weaknesses within the visuospatial domain that emerged in both participants speaks, in particular, of the multifaceted nature of spatial cognition and the divergent effects of genetic deletions within the WSCR on different components of visuospatial ability.

A deletion in HR of over 24 genes on the WSCR, including haploinsufficiency for *GTF2IRD1*, did not result in a phenotypic expression typically observed in WS nor in typical development. As such, the retention of the more telomeric genes in HR likely contributes to relatively good large-scale spatial cognition, albeit with atypical features. In JB, an individual with a contrasting deletion in the distal WS region from within *GTF2I* presented with good performance on mental rotation and VPT, alongside poor performance on large-scale spatial and general cognitive functioning measures. As such, these findings indicate that even in the face of a stark intellectual deficit as seen in JB, mental rotation abilities in particular are not affected by genes extending telomerically from *GTF2I*. Indeed, although conclusions cannot be met from such case studies regarding precise genotype-phenotype mapping, the current study provides further insight into the complex, dynamic, and combinatorial role of different genes within the WSCR on disparate phenotypic expression within the visuospatial domain.

## Abbreviations

aCGH: array comparative genome hybridisation; BAS-II: British Abilities Scale-II School Age; ELN: elastin; FISH: Fluorescent *in situ* Hybridisation; GTF: general transcription factor; LIMK1: Lim-kinase1; Mb: megabase; MR: mental rotation; RCPM: Ravens Coloured Progressive Matrices; SVAS: supravalvular aortic stenosis; TD: typically developing; VPT: visual perspective-taking; VE: virtual environment; WPPSI-R: Wechsler Preschool and Primary Scale of Intelligence- Revised; WS: Williams syndrome; WSCR: Williams syndrome critical region.

## Competing interests

The authors declare that they have no competing interests.

## Authors’ contributions

HB, EKF, and AK-S conceived of the study, participated in the design, data collection and analysis, and manuscript presentation; EC contributed to the conception, ran control participants, and design of the study and manuscript preparation; KM, MT, FS and PT contributed to the genetic analysis and participant recruitment and participated in manuscript preparation; EM contributed to interpretation of genetic analyses, preparation of the manuscript and Figure. All authors read and approved the final manuscript.
